# Tooth Root-Derived Graft Promotes Complete Bone Replacement in Alveolar Ridge Preservation: Comparative Study with a Collagenic Xenograft in Dogs

**DOI:** 10.3390/jfb17020077

**Published:** 2026-02-05

**Authors:** Yasushi Nakajima, Takahisa Iida, Elio Minetti, Maria Permuy, Giuliano Roberto, Ermenegildo Federico De Rossi, Giovanna Iezzi, Daniele Botticelli

**Affiliations:** 1 Department of Oral Implantology, Osaka Dental University, 8-1 Kuzuhahanazonocho, Hirakata, Osaka 573-1121, Japan; y.nakajima@me.com (Y.N.); iidataka@iris.ocn.ne.jp (T.I.); 2 ARDEC Academy, 47923 Rimini, Italy; e.f.derossi@gmail.com; 3 Department of Biomedical, Surgical, and Dental Science, University of Milan, 20122 Milan, Italy; elio.minetti@gmail.com; 4 Ibonelab, CEI Nodus, 27003 Lugo, Spain; maria.permuy@usc.es; 5 Department of Veterinary Clinical Sciences, Facultade de Veterinaria, Terra Campus, Universidade de Santiago de Compostela, 27002 Lugo, Spain; 6 Private Practice, 00072 Ariccia, Italy; dottgiulianoroberto@libero.it; 7 Department of Medical, Oral and Biotechnological Sciences, University G. D’Annunzio of Chieti, 66100 Chieti, Italy; gio.iezzi@unich.it

**Keywords:** bone substitutes, dentin, xenografts, bone regeneration, tooth extraction, alveolar ridge augmentation, dogs, histological techniques

## Abstract

Background: Autogenous tooth-derived grafts have been proposed as an alternative to xenografts for alveolar ridge preservation, offering biological similarity to bone and potentially more favorable remodeling. This study compared the healing outcomes of a collagenated xenograft, and a tooth-derived graft prepared with an automated processing device. Methods: Six Beagle dogs underwent bilateral extraction of the third and fourth mandibular premolars. Each animal contributed two sockets grafted with root-derived particulate prepared using an automated device for tooth cleaning, grinding, and demineralization, and two sockets grafted with a collagenated xenograft, all covered by a collagen membrane. After 3 months, histological sections were analyzed to assess crestal dimensions and the relative proportions of mature (lamellar) and immature bone (woven/parallel fibered), residual graft material, and soft tissues. Results: Lingual crest height did not differ between groups, whereas the buccal crest was slightly higher at xenograft sites compared with the tooth-graft sites. The tooth-graft group exhibited significantly fewer residual particles (0.5 ± 1.1%) and a higher proportion of total bone (65.6 ± 9.1%) compared with the xenograft group, which showed 19.7 ± 16.0% graft remnants (*p* = 0.032). Corticalization at the socket entrance was observed predominantly in the tooth-graft sites. No inflammatory infiltrates were detected in the examined section. Conclusions: Tooth-derived grafts promoted an almost complete replacement by vital bone with minimal residual material, whereas xenografts provided slightly better buccal contour preservation but resulted in regenerated tissues containing persistent graft particles. The biological differences observed may have implications for subsequent implant placement.

## 1. Introduction

The extraction of a tooth triggers processes that physiologically lead to bone resorption of the alveolar-supporting structure [1]. The volumetric reductions following tooth extraction were measured and ranged from 1.67 to 2.03 mm vertically and 3.87 mm horizontally [2]. Alveolar bone resorption occurs mainly during the first year [3].

More than 40 years ago, Dahlin suggested the use of a membrane to guide biological processes by excluding soft tissues from bone defects during regeneration [4]. It has also been proposed to use bone substitutes in particulate form to maintain space during healing.

Bone grafting materials currently used for alveolar ridge preservation include autografts, allografts, xenografts, and alloplastic substitutes, each characterized by distinct biological and remodeling properties. Among these, xenografts are widely employed due to their availability and volume stability, although their slow resorption may result in persistent residual particles [5,6].

Ridge preservation procedures may contribute to maintaining the volume and increasing the possibility of implant placement. Numerous animal studies have been conducted to investigate the biological behavior of grafting materials [7,8]. The placement of various autogenous, allogenic, or xenogeneic biomaterials in extraction sites has been extensively analyzed from both clinical and histomorphometric perspectives [9,10]. In a meta-analysis evaluating regenerated bone and preserved volumes, no biomaterial was found to be superior to the others for the analyzed parameters [11].

The latest innovation in graft materials consists of collagen coating, which has been proposed to facilitate cellular adhesion and biomaterial resorption [12,13,14,15,16]. This approach aims to overcome the limited resorption of some biomaterials, which may delay their replacement by newly formed bone while acting primarily as a scaffold for regeneration [17,18].

Due to its composition, the tooth has been considered a potential graft material with osteoinductive and osteoconductive properties. Dentin consists of approximately 65% hydroxyapatite, ten times denser than that found in bone, and 35% organic components, mainly type I collagen and proteins identical or similar to those present in bone [19,20,21,22]. The higher density of tooth-derived materials compared to bone may result in a slower resorption rate, which could be advantageous during bone healing by supporting gradual remodeling and replacement [23].

More than 50 years ago, the osteoinductive properties of dentin were demonstrated by the formation of bone tissue following implantation of dentin granules into muscle tissue in animal models [24,25]. More recent studies have identified the presence of bone morphogenetic protein-2 (BMP-2) in teeth, which promotes osteoblastic differentiation by inducing mesenchymal cell commitment [26].

Numerous studies have investigated the clinical outcomes of tooth-derived grafts produced using chairside devices [27,28,29,30]. One medical device is fully automated in the fragmentation, decontamination, and demineralization of teeth, producing an autologous biomaterial that can be used immediately in the same patient [31,32]. The clinical performance of this material has been evaluated in multiple clinical studies, including multicenter investigations conducted over time [33,34]. However, a histological comparison between extraction sockets filled with a graft derived exclusively from the tooth root (dentin-based particulate) and those treated with a collagenated xenograft has not yet been performed. Therefore, the aim of this study was to compare, in an animal model, the healing outcomes of socket preservation using a collagenated xenograft or a tooth root graft processed with an automated tooth-transforming device.

## 2. Materials and Methods

### 2.1. Ethical Statement

All experimental procedures were carried out in accordance with the European Directive 2010/63/EU concerning the protection of animals used for scientific purposes. The protocol was reviewed and approved by the Ethical Committee of the Rof Codina Foundation and by the regional authority (Xunta de Galicia, Xefatura Territorial da Consellería do Medio Rural, Ronda da Muralla 70, Lugo, Spain) under authorization code 01/20/LU-001 of 8 April 2020. The study was also performed in accordance with the ARRIVE reporting standards (The ARRIVE Essential 10: Compliance Questionnaire). Following the resolution of logistical constraints, the experiment was initiated in June 2023 and conducted within the five-year validity period of the ethical approval.

### 2.2. Experimental Animals

Six adult female Beagle dogs, approximately six years old and weighing on average 11.5 kg, were used in this experiment. The animals were obtained from Isoquimen (Barcelona, Spain) and underwent a three-week quarantine before the start of the study. All experimental procedures and housing were carried out at the CeBioVet research facility in Lugo, Spain.

### 2.3. Experimental Design

Following bilateral extraction of the third and fourth mandibular premolars, the mesial sockets were grafted with either a xenogeneic or an autologous tooth-derived material. Both sites were covered with a collagen membrane, and two sockets were treated with each graft type. Animals were euthanized after a three-month healing period, which was selected to allow evaluation of early to intermediate bone regeneration and graft remodeling.

### 2.4. Sample Size

No previous quantitative data were available on the expected differences in the primary outcome (vertical discrepancy between lingual and buccal bone peaks after healing) between collagenated xenograft and tooth root graft in post-extraction sockets in dogs. Therefore, an a priori theoretical sample size calculation was performed using G*Power (version 3.1.9.7, Universität Düsseldorf, Düsseldorf, Germany), assuming a repeated-measures design with two within-animal treatment conditions (collagenated xenograft and tooth root graft), an alpha level of 0.05, a power of 80%, and a large effect size as the minimum biologically relevant difference (Cohen’s dz ≈ 1.0 for paired comparisons). Under these assumptions, the required number of animals was six.

To comply with the 3R principle, each dog contributed four post-extraction sockets, two randomly allocated to the collagenated xenograft and two to the tooth root graft, resulting in a total of 24 experimental sites (12 per group), while keeping the number of animals as low as reasonably possible.

### 2.5. Randomization, Allocation Concealment, and Blinding Procedures

Randomization was performed digitally by one investigator (DB) who did not participate in the surgical phase. The treatment assignments were enclosed in opaque, sealed envelopes labeled with identification codes and kept by the same investigator, to be opened only after tooth extraction. All histological sections were coded to maintain blinding during the analysis, avoiding any identification of the experimental group.

### 2.6. Characteristics of the Biomaterials Used

OsteOXenon (BIOTECK, Arcugnano, VI, Italy): bone grafts consisting of cortical-cancellous natural equine bone granules (particle size: 0.25–1.0 mm), enzyme-deantigenated with preserved bone collagen.

Heart (BIOTECK, Arcugnano, VI, Italy): resorbable collagen membrane made of natural equine pericardium (20 × 20 mm in size; thickness 0.2–0.4 mm).

Tooth root graft: a graft material composed of root granules obtained through fragmentation, demineralization, and detoxification using the Tooth Transformer automated device (Tooth Transformer srl; Milan, Italy). Demineralization consists of a superficial reduction in the inorganic component of dentin, while detoxification ensures the elimination of organic residues and potential bacterial contamination. The processed granules retain their natural content of hydroxyapatite, collagen, and non-collagenous proteins. The treatment increases surface wettability and promotes cell adhesion through exposure of collagen fibers and enlargement of dentinal tubules [31,32,35].

### 2.7. Equipment for Tooth Graft Production

**Figure 1:**
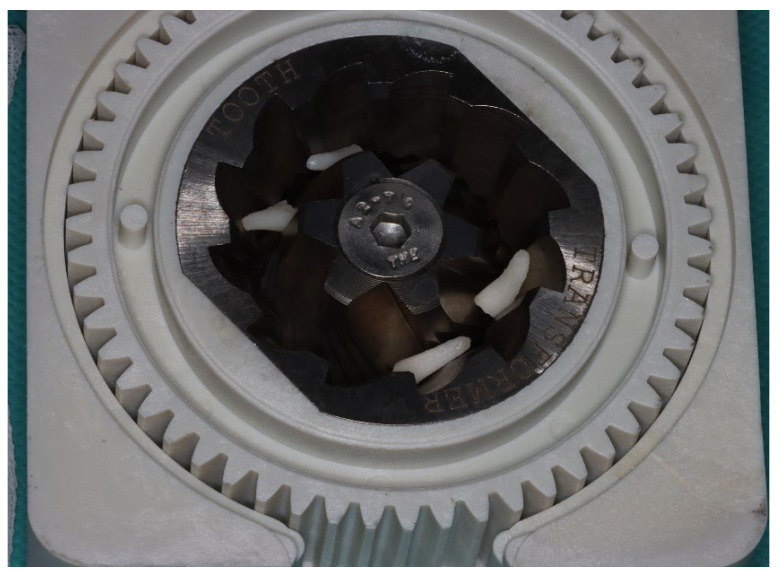
In this image, you can observe the dental residues placed inside the tooth transformer low-speed grinder. This grinder is made up of three multifunctional, autoclavable components, where the concentric conical blades, made in surgical steel, allow the production of granules of uniform size.

Autogenous tooth root graft was prepared using an automated processing unit (Tooth Transformer; Tooth Transformer srl, Milan, Italy; Figure 1).

Following extraction, the clinical crown was removed using a high-speed diamond bur under copious irrigation with sterile saline solution to avoid thermal alteration of the substrate. The remaining root was then sectioned into standardized fragments (approximately 1–3 mm) using sterile cutting instruments.

These fragments were placed into the device that produces the graft material (Figure 1). The tooth pieces were introduced into the device’s grinder, after which the single-use unit was opened and a cartridge containing the disposable processing solutions was inserted in the correct position. Once all components were assembled and the machine’s cover was closed, the device was activated using the main power switch.

The grinding unit consists of conical surgical-steel blades operating with factory-preset, non-modifiable parameters. The operator cannot adjust rotational speed, cutting time, or granulometry, as the entire grinding and processing sequence is fully automated. This design ensures procedural standardization and reproducibility across all samples (for details see [31]). The demineralized tooth graft material was ready after approximately 25 min from tooth extraction and was immediately placed into the extraction sockets during the same surgical session. The time interval between tooth extraction, graft preparation, and graft placement was therefore controlled and limited to the duration of the automated processing cycle. The processing workflow is standardized, as the device is fully automated and consistently reproduces the same sequence of steps, ensuring high procedural reproducibility.

### 2.8. Anesthesia Procedures

Prior to surgery, the dogs received intramuscular premedication consisting of medetomidine (10 µg/kg; Sededorm^®^ 1 mg/mL, Vetpharma Animal Health, Barcelona, Spain) and morphine (0.3 mg/kg; Morfina Braun 2%, B. Braun Medical, Barcelona, Spain). General anesthesia was induced with intravenous propofol (2 mg/kg; Propofol Lipuro^®^ 10 mg/mL, B. Braun, Melsungen, Germany) and maintained with isoflurane at 1–1.5% in oxygen (Vetflurane^®^ 1000 mg/g, Virbac SA, Carros, France). Physiological parameters including heart rate, respiratory rate, arterial pressure, and end-tidal CO_2_ were continuously monitored throughout anesthesia. Measurements were recorded every 5 min during induction and subsequently at 15 min intervals.

### 2.9. Surgical Procedures

During the initial surgical session, a full-thickness flap was meticulously raised at the 3rd and 4th premolars bilaterally. The teeth were hemisected and extracted. The remaining portion of the crown was eliminated and only the roots were used for the tooth graft production to ensure that no enamel portion remained. The alveoli were randomly filled with either xenograft or tooth graft and subsequently covered with a collagen membrane without the use of any fixation procedure. Semi-submerged closure was achieved using interrupted 4-0 Vicryl sutures.

### 2.10. Housing and Husbandry

Animal well-being was monitored daily by a trained veterinarian through standardized clinical observation, including assessment of general behavior, food intake, wound appearance, swelling, and signs of discomfort or pain. No clinical signs requiring additional analgesic or anti-inflammatory treatment were observed.

During the postoperative healing period until euthanasia, all animals received a standard commercial dog diet softened with water, in order to reduce mechanical stress on the extraction and grafted sites during healing.

Postoperative analgesia consisted of intramuscular buprenorphine (0.01 mg/kg every 8 h; Bupaq, Richter Pharma AG, Wels, Austria). Meloxicam (Loxicom, Norbrook Laboratories, Monaghan, Ireland) was given intravenously before surgery (0.2 mg/kg) and subsequently administered orally for two consecutive days (0.1 mg/kg once daily). Antimicrobial prophylaxis included intravenous cefazolin (20 mg/kg; Cefazolina Normon, Madrid, Spain) and subcutaneous sodium cefovecin (8 mg/kg; Convenia, Zoetis, Madrid, Spain) administered prior to each surgical session.

Post-surgical wound care included daily inspection of the surgical sites during the first week, followed by cleaning with gauze moistened with 0.12% chlorhexidine solution three times per week until complete soft-tissue healing was achieved. No adverse events or complications were observed during the postoperative period.

### 2.11. Euthanasia

Three months after the final surgical procedure, the animals were sedated and humanely euthanized by intravenous administration of an overdose of sodium pentobarbital (200 mg/kg; Dolethal, Vetoquinol, France).

### 2.12. Histological Preparation

After fixation in buffered 10% formaldehyde, the tissue specimens were dehydrated through a graded series of ethanol solutions and subsequently embedded in a light-curing resin (Technovit 7200 VLC; Heraeus-Kulzer GMBH, Wehrheim, Germany). Radiographic imaging was used to identify the region of interest and guide sectioning along the buccolingual plane with a precision band saw (Exakt Apparatebau, Norderstedt, Germany). The obtained sections were ground and polished to a final thickness of approximately 50 µm. Histological slides were prepared and stained using the Levai–Laczkó technique.

### 2.13. Clinical Evaluation

After tooth extraction, measurements were taken with a UNC probe to determine the buccolingual width of the socket and the vertical levels of the buccal and lingual bone crests relative to the socket floor. The buccal bone thickness was measured 1 mm apical to the crest using an Iwanson caliper (Koiné Italia snc, Milano, Italy).

### 2.14. Histological and Morphometric Analysis

Histological evaluations were performed using an Eclipse microscope (Nikon Corporation, Tokyo, Japan) connected to a computerized imaging system. Quantitative assessments were carried out with NIS-Elements D 5.11.00 software (Laboratory Imaging, Nikon Corporation, Tokyo, Japan).

**Figure 2:**
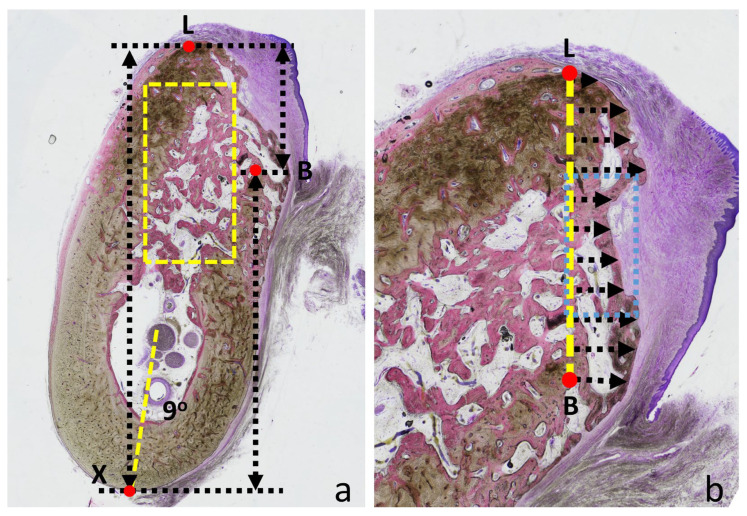
(**a**) Assessments were performed by positioning the mandibular base axis at a 9° angle relative to a vertical plane (yellow dashed line). The boundaries at the bone crest separating pre-existing bone from newly formed bone are indicated as L (lingual aspect) and B (buccal aspect). X represents the apical point of the mandibular base. The distances between L and X (L–X) and between L and B (L–B) were measured. The yellow dashed rectangular area indicates the region selected for morphometric analysis. (**b**) The line connecting L and B was subdivided into ten equal intervals, and at each point, the perpendicular distance to the outline of the newly formed bone was recorded (black dotted arrows). Light blue dashed rectangle: area of morphometric analysis.

To minimize potential measurement bias, the histological images were analyzed in different orientations. A reference angle of 9° from the vertical plane of the mandibular base was adopted to reproduce the natural spatial inclination of the mandible, as applied in a previous animal study (Figure 2a) [36].

The following histological assessments were performed:

1. The boundaries separating the pre-existing bone and the newly formed bone were identified at both the lingual (L) and buccal (B) aspects, following the method described by Araújo et al. (2005) [1]. The distances between the apical reference point of the mandible (X) and the points L and B were measured. The difference between these two values was expressed as the L–B parameter.
2. A rectangular region of interest (ROI), 3 mm wide and 6 mm deep, was superimposed on each histological section, maintaining the 9° inclination (yellow dashed rectangle). The rectangle was positioned to simulate a biopsy retrieval along the ideal axis for dental implant placement, and quantitative assessments were performed within this region. Mature bone (lamellar bone), immature bone (woven/parallel-fibered bone), residual graft material, and soft tissues were identified according to established histological criteria, and their relative proportions were calculated.
3. A straight-line connecting points L and B was traced, and the histological image was oriented so that the L–B line was aligned vertically (Figure 2b). This L–B line was subdivided into ten equal segments using a superimposed grid. At each division point, the most peripheral bone extension beyond this line was measured along the grid boundaries.
4. A vertically aligned rectangular ROI (1 mm × 2 mm) was superimposed on each histological section at the coronal aspect of the alveolus, and quantitative assessments were performed within this region (light blue dashed rectangle; Figure 2b). Mature bone (lamellar bone), immature bone (woven/parallel-fibered bone), residual graft material, and soft tissues were identified according to established histological criteria, and their relative proportions were calculated.

### 2.15. Data Analysis

Histometric measurements were performed by a calibrated examiner (EFDR) under the supervision of an experienced investigator (DB). Although each dog provided four post-extraction sockets (two treated with collagenated xenograft and two with tooth root graft), the animal was considered the primary experimental unit. For the main analysis of the primary outcome, the vertical discrepancy between lingual and buccal bone peaks was first averaged at the dog level for each treatment (mean of the two xenograft sites and mean of the two tooth root graft sites per dog).

Data distribution was assessed using the Shapiro–Wilk normality test. Depending on the outcome, either a paired *t*-test or a Wilcoxon matched-pairs signed-rank test was applied for statistical comparison. The significance level was set at α = 0.05.

The primary outcomes variables were L-B and total bone percentage within the ROI. The software GraphPad Prism (v. 10.6.1, GraphPad Software, San Diego, CA, USA) was used for the statistical analyses. For each parameter, mean values from the two socket sites were calculated, and the resulting data were analyzed at the animal level.

## 3. Results

### 3.1. Clinical Evaluations

No anesthesia-related adverse events or surgical complications, such as excessive bleeding, wound dehiscence, membrane exposure, infection, or signs of local inflammation, were observed during the postoperative period. Of the 24 extraction sockets originally prepared (four per animal), 23 were available for analysis. One site was eliminated because a fracture of the buccal bone occurred after tooth extraction, and the corresponding measurements and histologic assessment could not be reliably collected. The remaining sites consisted of 12 sockets grafted with tooth root graft and 11 with collagenated xenograft, maintaining a balanced allocation across the animals.

The dimensional characteristics recorded immediately after extraction were comparable between the two groups (Table 1).

Buccal depths measured 8.2 ± 0.8 mm in tooth root grafted sites and 8.3 ± 0.7 mm in xenograft sites, while lingual depths were 8.5 ± 0.6 mm and 8.7 ± 0.8 mm, respectively. The bucco-lingual and mesio-distal diameters also showed no differences, and the buccal bone wall was consistently thin (approximately 0.7–0.8 mm) in both groups. These findings confirm that the two sets of sockets were equivalent at baseline.

### 3.2. Histological Qualitative Evaluation

After 3 months of healing, both the xenograft and tooth root graft groups showed newly formed bone within the alveolar sockets. No inflammatory infiltrates or multinucleated giant cells were detected in any of the examined sections in both groups.

**Figure 3:**
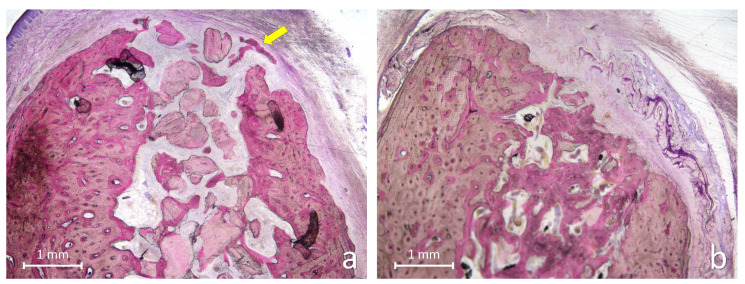
Photomicrographs of ground sections illustrating the healing after 3 months at xenograft sites. (**a**) A large number of graft granules can be observed, surrounded by newly formed bone and soft tissues. A small portion of new bone is evident at the periphery of the coronal aspect of the alveolus bridging graft particles (yellow arrow). (**b**) A substantial amount of newly formed bone relative to residual graft granules was observed in this alveolus. The coronal entrance of the alveolar socket appears nearly completely sealed by new bone formation. Remnants of the collagen membrane are still visible. Levai–Laczkó stain.

At the xenograft sites, the internal portion of the alveoli was in some specimens largely occupied by large amount of residual graft granules (Figure 3a) while other alveoli presented larger region with newly formed bone intermingled with residual graft granules (Figure 3b).

**Figure 4:**
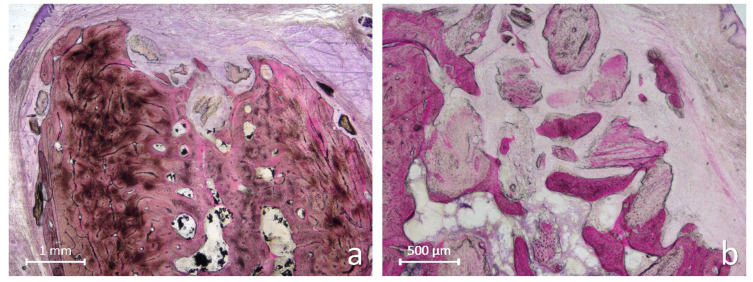
Photomicrographs of ground sections illustrating the healing after 3 months at xenograft sites. (**a**) The internal region of the alveolus appears filled with newly formed bone, whereas the coronal region contains several xenograft granules that interfere with complete healing. (**b**) Another example of poor healing observed at the coronal entrance of the alveolar socket. Levai–Laczkó stain.

The newly formed bone exhibited irregular trabeculae in close contact with the granule surfaces. In the coronal region, numerous xenograft granules remained embedded within fibrous connective tissue, often interfering with the complete closure of the alveolar entrance (Figure 4a,b).

**Figure 5:**
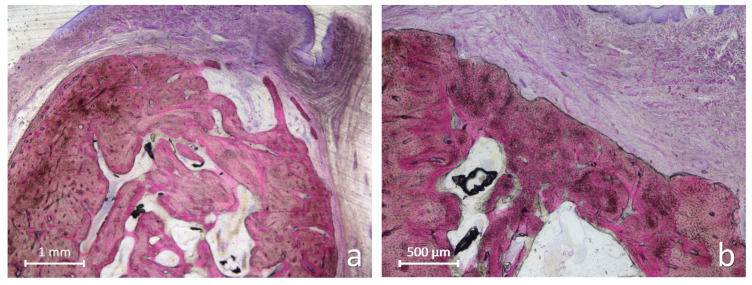
Photomicrographs of ground sections illustrating the healing after 3 months at tooth root graft sites. (**a**) The alveolus appeared filled with new bone presenting regions with advanced bone maturations. The coronal portion of the alveolus showed intertrabecular spaces filled with immature fibrovascular tissue, clearly separated from the overlying mucosal connective tissue. (**b**) The entrance of the alveolus appears closed by mature, corticalized bone. Levai–Laczkó stain.

At the tooth root graft sites, the alveoli were predominantly filled with newly formed bone, in several areas showing advanced maturation and early corticalization, especially in the coronal region (Figure 5a).

**Figure 6:**
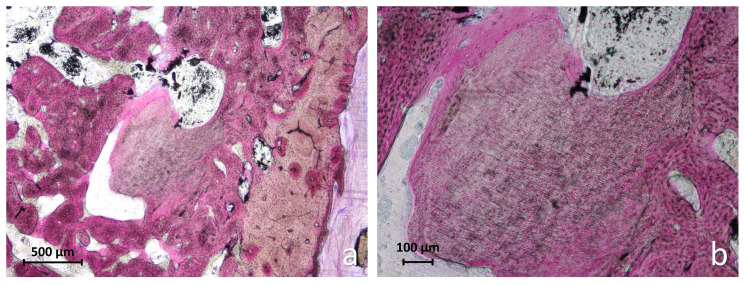
Photomicrographs of ground sections illustrating the healing after 3 months at tooth root graft sites. (**a**) A small residual graft granule was observed within the alveolus, surrounded by newly formed bone. (**b**) Higher magnification of the region of interest shown in (**a**) revealed a structure resembling a dentin remnant. Levai–Laczkó stain.

The intertrabecular spaces in the coronal portion were filled sometimes with immature fibrovascular tissue, clearly distinct from the overlying mucosal connective tissue. In most specimens, the coronal entrance of the socket appeared completely sealed by mature, corticalized bone (Figure 5b). Only one residual granule resembling dentin remnants was observed, surrounded by newly formed bone (Figure 6a,b).

### 3.3. Histological Quantitative Evaluation

Quantitative data are presented as mean values with associated variability to reflect intra-group heterogeneity.

Because the clinical discrepancy between the lingual and buccal crest depths was negligible (0.1 mm), the histological measurements were considered reliable, and no correction factors were applied.

After three months of healing, the lingual crest height (L–X) showed identical mean values in the two groups (14.8 ± 1.8 mm). The buccal crest (B–X) exhibited a small but statistically significant difference, measuring 11.8 ± 1.7 mm in tooth root grafted sites and 12.3 ± 1.8 mm in xenograft-treated sites (*p* = 0.031). The resulting vertical discrepancy between the lingual and buccal crestal peaks (L–B) was 3.0 ± 1.2 mm for the tooth root graft group and 2.5 ± 0.5 mm for the xenograft group, with no statistically significant difference (Table 2).

Histomorphometric analysis revealed distinct tissue compositions. Tooth-grafted sites demonstrated a high proportion bone, with 34.1 ± 13.0% immature bone and 31.5 ± 16.1% mature bone, for a total bone fraction of 65.6 ± 9.1%. Residual graft material was almost absent (0.5 ± 1.1%), and soft tissues accounted for 33.9 ± 9.4%. In xenograft sites, the total bone fraction was lower (57.8 ± 17.2%), composed of 32.1 ± 11.7% immature bone and 25.7 ± 6.7% mature bone, while a substantial amount of graft remnants was still present (19.7 ± 16.0%). The difference in residual biomaterial between the two groups was statistically significant (*p* = 0.032; Table 3).

**Figure 7:**
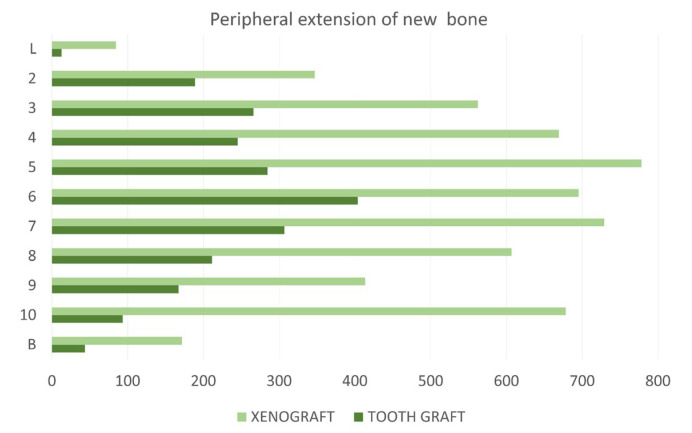
Graph showing the most peripheral bone extension beyond the vertical line drawn between L and B measured at each line delimiting the squares of the grid. Measures reported in µm.

The most peripheral bone extension beyond the L–B line was greater in the xenograft group (0.52 ± 0.23 mm) compared with the tooth graft group (0.20 ± 0.12 mm; *p* < 0.0001; Figure 7).

Quantitative histomorphometric analysis of the coronal region is summarized in Table 4. In the tooth root graft group, the coronal region was characterized by a higher proportion of total bone (59.8 ± 14.8%) compared with the xenograft group (42.3 ± 16.2%), although this difference did not reach statistical significance (*p* = 0.239).

The proportions of mature and immature bone did not differ significantly between groups (*p* = 0.317 and *p* = 0.269, respectively). No graft material was observed in the tooth root graft group, whereas xenograft-treated sites showed a measurable amount of residual graft in the coronal region (20.3 ± 16.5%), with a statistically significant difference between groups (*p* = 0.044). No significant differences were observed in the proportion of soft tissue (*p* = 0.562).

## 4. Discussion

### 4.1. Overview of the Main Findings

Healing proceeded uneventfully in all animals, and the two grafting materials produced distinct patterns of ridge remodeling. At baseline, the extraction sites showed comparable dimensions between the groups, with similar buccal and lingual depths, socket diameters, and buccal wall thicknesses. This uniformity confirms that the differences observed after three months reflect the biological behavior of the materials rather than anatomical variations at the start of the experiment.

At the end of the healing period, the lingual crest height was identical in the two groups, suggesting that the lingual aspect remained relatively stable regardless of the grafting material or was subjected to similar remodeling process. As expected, the main changes occurred at the buccal aspect, with the xenograft group showing a slightly higher crest than the tooth root graft group.

When the discrepancy between lingual and buccal peaks was considered, a tendency was observed toward a slightly greater vertical difference in the tooth root graft group. Although this difference did not reach statistical significance, it suggests two distinct modes of healing: one driven predominantly by bone formation (tooth root graft) and one partly supported by the persistence of residual biomaterial particles (xenograft).

### 4.2. Histological and Biological Interpretation

The histomorphometric analysis provides a clearer interpretation of these dimensional outcomes. Sites grafted with tooth-derived particles showed a high proportion of bone, approximately two-thirds of the tissue present, combined with an almost complete absence of residual graft.

In contrast, the xenograft sites showed a slightly lower percentage of total bone compared with the tooth-graft group, and a substantial amount of graft remnants, accounting for nearly one-fifth of the total tissue volume. These findings align closely with human clinical studies in which tooth-derived materials were associated with limited residual graft and high vital bone percentages [37].

Another aspect that deserves consideration is that the higher total bone content observed in the tooth root graft group was largely attributable to a greater proportion of mature bone. Part of this mature bone corresponds to pre-existing cortical bone located on the lingual side of the socket, which was included within the ROI in both groups. However, because this anatomical component was present to the same extent in both grafting conditions, between-group difference in mature bone is consistent with a trend toward faster maturation of the newly formed bone in the tooth-graft sites compared with the xenograft sites. Nevertheless, none of the differences in the percentage of mature, woven, or total bone reached statistical significance.

From a biological perspective, the near-complete replacement of the tooth root graft by vital bone may be explained, at least in part, by the intrinsic composition of dentin, which includes type I collagen, non-collagenous proteins, and growth factors that are preserved by the demineralization process [22].

These characteristics may support early osteoblastic activity and favor more rapid turnover of the graft. Xenografts, on the other hand, provide an osteoconductive scaffold with slower resorption kinetics. Their persistence in the healing site can contribute to ridge preservation but also results in regenerated tissues containing both new bone and biomaterial particles. In the present study, this corresponded to slightly better vertical maintenance at the buccal aspect in the xenograft group.

### 4.3. Vital Bone Regeneration vs. Hybrid Bone Regeneration

Consistently with the histomorphometric findings described above, both materials produced favorable outcomes, albeit with different biological implications. Tooth root grafting resulted in regenerated sites composed almost entirely of vital bone with virtually no residual biomaterial, whereas xenografts yielded a more stable buccal profile but contained a substantial proportion of long-lasting particles, leading to the formation of a hybrid-type bone.

The clinical relevance of these differences remains to be fully clarified, but the composition of the regenerated site may influence later remodeling stages and implant osseointegration. Importantly, several of the observed histological differences were qualitative in nature and did not reach statistical significance.

Nevertheless, it is worth noting that the use of slowly resorbing biomaterials has been associated with impaired osseointegration of implants placed six months after sinus floor elevation [38]. Residual xenograft particles and degradation products remained in close contact with the implant surface even after three months of healing, limiting the progression of new bone formation and preventing complete osseointegration in the areas where graft remnants persisted.

Under this concept, the presence of sound new bone may be considered biologically favorable compared with a hybrid bone containing residual, non-resorbed biomaterial, although this interpretation remains speculative and was not directly assessed in the present study [38].

### 4.4. Coronal Maturation and Corticalization

In the coronal region of the alveolus, sites grafted with tooth-derived material showed a higher proportion of bone compared with xenograft-treated sites. However, this difference did not reach statistical significance (*p* = 0.239) and was associated with considerable intra-group variability. Therefore, the observed difference should be interpreted as a trend rather than as evidence of a definitive effect.

Another relevant observation is the presence of a relevant proportion of graft material in the xenograft group. Because such sites are typically regenerated to allow predictable implant placement, the persistence of non-resorbed graft particles in the most coronal portion may potentially hinder osseointegration in one of the most delicate regions of the implant, although implants were not placed in the present study.

At the same time, xenografts tended to preserve the buccal contour slightly better than tooth root grafts, suggesting that volume stability and biological quality do not necessarily coincide. This consideration opens the possibility of combining these advantages, namely, improved corticalization and enhanced ridge preservation, through the adjunctive use of a cortical lamina placed on the buccal surface.

### 4.5. Emerging Strategies for Alveolar Ridge Preservation

In recent years, alternative strategies have been proposed to improve ridge maintenance and promote the formation of vital bone following tooth extraction. Among these, approaches based on the temporary separation of the periosteum from the underlying buccal bone surface have suggested that reducing periosteal contact might attenuate post-extraction remodeling. In this context, the interposition of thin barriers—such as high-density PTFE membranes or cortical laminae—between the buccal bone plate and the elevated flap has been proposed as a means of mechanically shielding the buccal bone during early healing [39,40,41].

### 4.6. Biological Safety and Biocompatibility of the Tooth Root Graft

A further aspect that deserves consideration is related to the nature and handling of the graft material. In this study, the graft was prepared from the animals’ own extracted teeth, after removing the crowns and enamel, and the initial phases of manipulation were performed under non-sterile conditions. Sterility was re-established only after the particulate was introduced into the processing device, where decontamination and preparation occurred in a fully controlled environment. Despite these non-sterile preliminary steps, the sites grafted with tooth-derived material healed uneventfully, and the histological sections showed no inflammatory infiltrates. This finding confirms both the biocompatibility of the autogenous tooth root matrix and the effectiveness of the device’s decontamination and preparation protocol, which produced a sterile and clinically safe graft confirming the results from other studies in humans [34,37]. Although exploratory in nature, the coherence between the clinical measurements and histological findings supports the validity of the observations. The consistency of our results with those from human studies further reinforces the biological plausibility of the healing pattern associated with tooth-derived grafts.

### 4.7. Methodological and Biological Strengths

The main strength of this study lies in its within-animal design, which minimizes biological variability and allows a reliable comparison between the two grafting materials under identical healing conditions. The assessment of the histological composition also provides a comprehensive view of the regenerative process, highlighting not only differences in ridge preservation but also in the biological quality of the newly formed tissues. In the tooth root graft group, this was associated with regenerated tissue largely composed of vital bone and showing clear corticalization at the coronal entrance, whereas in the xenograft group a hybrid bone was observed, characterized by the presence of numerous graft remnants. Another strength is the use of tooth-derived graft obtained from the animals’ own extracted teeth. While the preliminary handling of the teeth was performed under non-sterile conditions, the device’s processing cycle effectively re-established sterility, ensuring that the material placed into the sockets was fully decontaminated. The uneventful healing and lack of inflammatory infiltrates provide further confirmation of the graft’s biocompatibility and of the reliability of the decontamination protocol.

### 4.8. Study Limitations

Several limitations should be acknowledged in the present study. The number of animals was limited, in accordance with ethical principles, which reduced the ability to detect subtle differences between the two materials. The evaluation was limited to a single healing interval of three months, which reflects early to intermediate healing stages and does not allow assessment of long-term remodeling dynamics. Histological analysis was based on a single section per site, which does not capture the three-dimensional complexity of the regenerated tissues.

The absence of ungrafted control sockets represents an additional limitation, as it precludes direct comparison with spontaneous healing; however, the inclusion of further experimental groups would have reduced the statistical power of the within-animal comparison between grafted sites or required the use of additional animals.

The assessment of inflammatory response was limited to conventional histology, as no additional techniques such as immunohistochemistry were applied to detect low-grade or focal inflammatory infiltrates.

The present study did not include direct physicochemical characterization of the processed tooth graft. While such analyses have been previously reported for demineralized dentin matrices, their absence in the present work represents a limitation and should be considered when interpreting the biological findings [32].

Finally, although the dog model is widely accepted for alveolar ridge research, extrapolation to human clinical scenarios should be made with caution. Accordingly, the present findings should be regarded as hypothesis-generating and not as directly transferable to clinical protocols. Future studies incorporating longer follow-up periods, three-dimensional volumetric analyses (e.g., micro-CT), and implant-based validation models are required to better clarify the long-term biological and clinical relevance of these regenerative approaches.

## 5. Conclusions

Within the limits of this animal study, the use of tooth root–derived grafts resulted in regenerated tissues largely composed of vital bone, with minimal residual graft material, whereas xenografts were associated with a higher proportion of persistent particles while providing slightly better buccal contour preservation.

Both grafting approaches supported uneventful healing; however, the regenerated tissues differed mainly in their composition rather than in quantitatively proven bone outcomes, reflecting distinct biological remodeling patterns. These differences were largely qualitative in nature and should be interpreted with caution given the limited sample size and the absence of implant placement in the present model.

Further investigations incorporating region-specific analyses, three-dimensional volumetric assessments, and implant-based validation are required to better clarify the biological and clinical implications of these regenerative strategies.

## Figures and Tables

**Table 1:** Clinical measurements (in millimeters) of the extraction socket dimensions. Depth at the buccal (B) and lingual (L) aspects; bucco-lingual (B-L) and mesio-distal (M-D) diameters; thickness of the buccal bone wall; ≠L–B, difference between the lingual and buccal depths.

	Depth		Diameter		Thickness	≠Depth
	B	L	Bucco-Lingual	Mesio-Distal	Buccal	≠L-B
Tooth root graft	8.2 ± 0.8	8.5 ± 0.6	4.0 ± 0.3	4.3 ± 0.3	0.8 ± 0.1	0.3 ± 0.3
Xenograft	8.3 ± 0.7	8.7 ± 0.8	3.8 ± 0.3	4.2 ± 0.3	0.7 ± 0.1	0.4 ± 0.2
*p*-value	0.822	0.750	0.625	>0.999	0.125	>0.999

**Table 2:** Histological measurements (in millimeters). L and B indicate the points separating pre-existing bone from newly formed bone at the lingual (L) and buccal (B) crests, respectively; X represents the base of the mandible. L–X and B–X indicate the vertical distances between X and the lingual and buccal crests, respectively. ≠L–B is the difference between L–X and B–X.

	L–X	B–X	≠L–B
Tooth root graft	14.8 ± 1.8	11.8 ± 1.7	3.0 ± 1.2
Xenograft	14.8 ± 1.8	12.3 ± 1.8	2.5 ± 0.5
*p*-value	0.928	0.031	0.386

**Table 3:** Morphometric analyses expressed as percentages at the simulated biopsy retrieval site.

	Mature Bone	Immature Bone	Total Bone	Graft	Soft
Tooth root graft	31.5 ± 16.1	34.1 ± 13.0	65.6 ± 9.1	0.5 ± 1.1	33.9 ± 9.4
Xenograft	25.7 ± 6.7	32.1 ± 11.7	57.8 ± 17.2	19.7 ± 16.0	22.6 ± 6.5
*p*-value	0.393	0.830	0.277	0.032	0.058

**Table 4:** Quantitative morphometric analyses expressed as percentages in the coronal region of the alveoli.

	Mature Bone	Immature Bone	Total Bone	Graft	Soft
Tooth root graft	7.1 ± 8.3	52.7 ± 14.3	59.8 ± 14.8	0.0 ± 0.0	40.3 ± 14.8
Xenograft	5.3 ± 2.9	37.0 ± 15.9	42.3 ± 16.2	20.3 ± 16.5	37.5 ± 15.2
*p*-value	0.317	0.269	0.239	0.044	0.562

## Data Availability

The original contributions presented in the study are included in the article, further inquiries can be directed to the corresponding author.
